# Elucidation of contact state on various rough surfaces via highly robust colorimetric optical interferometry

**DOI:** 10.1038/s41598-021-04104-y

**Published:** 2022-01-07

**Authors:** Tatsunori Tomota, Mamoru Tohyama, Kazuyuki Yagi

**Affiliations:** 1 Powertrain System Research-Domain, Toyota Central R&D Labs., Inc., 41-1, Yokomichi, Nagakute, Aichi 480-1192 Japan; 2 Department of Mechanical Engineering, Faculty of Engineering, Kyushu University, 744, Nishi-ku, Fukuoka, Motooka 819-0395 Japan; 3 International Institute for Carbon-Neutral Energy Research (I2CNER), Kyushu University, 744, Nishi-ku, Fukuoka, Motooka 819-0395 Japan

**Keywords:** Optical techniques, Mechanical engineering, Imaging techniques, Surface patterning

## Abstract

In this study, we developed and practiced colorimetric optical interferometry for the direct observation of contact states to clarify contact phenomena. We theoretically demonstrated that the effect of roughness diffuse reflection could be neglected using interferometric light intensity according to the relationship between the optical film thickness and hue. Then, we measured the static contact surfaces of spherical test pieces of different root mean square roughnesses. Results indicate that the nominal contact area is significantly larger than that obtained from the Hertzian theory of smooth contact as the surface roughness increases. The contact film thickness on the nominal contact area increases almost in proportion to the root mean square roughness. Our experiment supports the validity of the contact theory and contact simulation with very small roughnesses, which have been difficult to verify experimentally. The advantage of this measurement is that it can simultaneously capture the macroscopic contact area and microscopic film thickness distribution, which is expected to further expand the range of application.

## Introduction

Friction phenomena caused by the contact between objects are extremely complex, and many attempts have been made to understand these phenomena from various perspectives. The Coulomb-Amontons law is a well-known empirical law, which states that the frictional force is proportional to the vertical load and independent of the nominal contact area^1,2^. The most common mechanism is as follows^3–5^. The apparently smooth surface of an object actually exhibits a complex roughness profile, and only a few “rough surface asperities” of this profile are in contact with the nominal contact surface, and such a “real contact area” is dependent on the load alone. Because shear forces are generated at the contact interface, the total frictional force is proportional to the real contact area. Although the above explanation is only valid under very limited conditions, such as dry friction or significantly low velocities, wherein the effects of intervening fluids can be neglected, it serves as the basis for understanding the phenomenon of friction.

As the real contact occurs at the roughness asperities, the surface roughness profile significantly influences the contact and friction phenomena. Differences in contact conditions due to roughness are also important in practical applications. For example, changes in the frictional force significantly impact energy loss, thereby affecting the performance of mechanical parts, and changes in the degree of wear directly affect product life and reliability. Furthermore, it is widely known from both empirical and theoretical predictions that a small gap exists on the contact surface of the joint in various sealed structures, and the amount of leakage from the gap varies depending on the surface finish^6–8^. Therefore, efforts have been made to improve the cutting process and optimize the surface finish to achieve an advantageous surface roughness for the product.

However, quantitative evaluation of the effect of surface roughness on contact phenomena has not yet been sufficiently conducted. For example, in the past, friction was thought to decrease with decreasing roughness. In reality, a large friction coefficient may be obtained on a rough surface with a considerably low roughness^9^. Currently, it is clear that the real contact area changes owing to the difference in the profile of the surface roughness, even if the surface roughness has the same size^10–12^. Although the influence of the surface profile can be measured by scalar values, such as the “real contact area” and “friction coefficient,” it is difficult to measure the actual contact state. Recently, direct simulations have been actively conducted using finite element methods; however, they are not often compared with the experimental results^13–15^.

The methods for observing the contact state can be broadly classified into direct measurement using optical approaches and indirect measurement using several other methods. Typical examples of the former are optical interferometry^16–20^ and laser scanning measurement^21,22^, both of which can visually capture the distribution of real contact points and gaps on the contact surface. However, both methods require one smooth contact surface comprising a light-transmitting material, such as glass. In particular, optical interferometry is limited to smooth surfaces with sufficiently low roughness in comparison with transmissive materials; thus, the practical observation target is almost always in the so-called “hydrodynamic lubrication” state, in which sliding occurs through inclusions, such as oil. Contrastingly, laser scanning is capable of capturing gaps caused by minute roughness with high resolution, which enables the detailed observation of the contact state even on surfaces with high roughness. However, because laser scanning is time-consuming, it is unsuitable for the real-time measurement of dynamic sliding surfaces. For indirect measurement, the electrical method using conductive materials^23–26^ is a typical example. This method can detect changes with high sensitivity because the amount of current is proportional to the radius of the contact circle; however, its value is limited to a scalar quantity. In this manner, each of the conventional measurement methods has advantages and disadvantages; therefore, a more convenient measurement method is required. In particular, a measurement method for the contact state of high-quality machined surfaces with a surface roughness (arithmetic mean roughness) of 10–100 nm is useful both academically and practically.

In this study, we aim to extend the application of optical interferometry to root mean square roughnesses of up to 100 nm, which has been considered to be difficult, and demonstrate on theoretical grounds that application is possible by using hues and three single-wavelength light sources. We also demonstrate that the newly proposed filtering method can convert the interference images with large variations into the gap distribution instantly. Then, using this new measurement method, we measure the gap size distribution of the contact surface in static contact of various spherical rough surfaces. Finally, we evaluate the effects of surface roughness and pressing load on contact conditions, such as average clearance and nominal contact area.

## Theory

**Figure 1:**
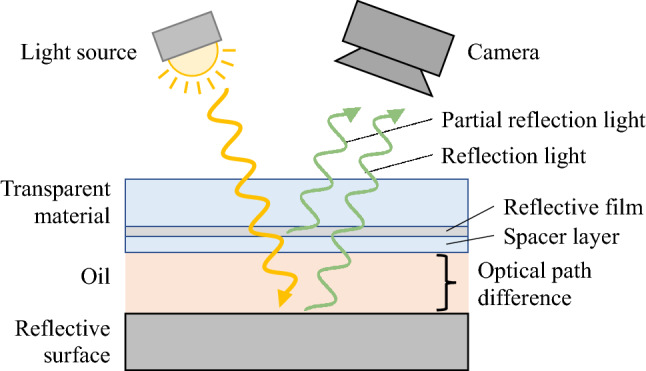
Concept of optical interferometry.

First, the measurement principle of colorimetric light interferometry^16–20^ is presented. In the measurement system shown in Fig. 1, the light emitted from the light source traverses the transparent material. A part of the light is reflected at the reflective film; the remaining light is transmitted through the spacer layer, and the oil is reflected by the reflective surface toward the direction of the light source. Because there exists an optical path difference between the light reflected at the front and back of the oil, an optical interference image is created, which is determined by the relationship between the optical path difference and the wavelength of the irradiated light. In this case, the interference luminance, $I$, is determined as follows.

1 $$I\left( t \right) = I_{1} + I_{2} + 2\sqrt {I_{1} I_{2} } \cos \left( {4\pi t_o/\lambda } \right).$$

Here, ${I}_{1}$ and ${I}_{2}$ are the front and back reflected light intensities, respectively; $\lambda$ is the incident light wavelength; and $t_o$ is the optical film thickness (the optical film thickness is the actual spatial distance multiplied by the refractive index of the oil). That is, the intensity of the interfering light varies sinusoidally with the thickness of the optical film, and its period and phase vary with the wavelength, such that complex interference fringes are formed according to the film thickness distribution. If the color information of the interfered light and the film thickness can be matched on a one-to-one basis, the distribution of the optical film thickness, i.e., the gap amount, can be obtained from the interference image captured by a camera coaxial to the incident light. In this case, the interferogram obtained by the camera is averaged with a resolution corresponding to the image resolution, and the brightness value of each pixel can be regarded as the average value of the optical film thickness in the pixel.

The above is the outline of the colorimetric interferometry, which is further classified according to the type of light source used and the method of conversion to the optical film thickness. The most common method is to use a white light source, wherein the relationship between the RGB or hue values of the interfering light acquired by the camera and the film thickness is determined in advance. This relationship is described by a calibration curve using a reference device, such as a precision steel ball, and then the calibration curve is used for another measurement target. This method is still commonly used because it can measure the film thickness between near-smooth surfaces with extremely high accuracy. However, the accuracy deteriorates significantly when the reflective surface is rough because, as the surface roughness increases, the ratio of diffuse reflection increases and the luminance of the backside light decreases. This occurs owing to the rapid decrease in specular reflectance as the length scale of the surface roughness approaches the wavelength of the monochromatic light. Assuming that the reflectance at the horizontal position $(x,y)$ is $R(x,y)$ and that the light absorption by the oil can be neglected owing to the small optical film thickness, ${t}_{o}$, Eq. (1) can be rewritten as follows.

2 $$I\left( t \right) = I_{1} \left\{ {1 + R\left( {x,y} \right)} \right\} + 2I_{1} \sqrt {R\left( {x,y} \right)} \cos \left( {4\pi t_{o} /\lambda } \right).$$

Because the reflectance $$R(x,y)$$ fluctuates depending on the position, the deviation from the calibration curve by the reference unit becomes large, making it unsuitable for high-resolution film thickness measurement. To address this problem, we adopted a combination of single-wavelength light sources^17,27^ instead of white light sources. A single-wavelength light source can be obtained by combining a monochromatic light source with a high-pass filter and a low-pass filter to cut off wavelengths of 5 nm or more before and after the specified wavelength. LEDs can also be used, as their performance has improved significantly in recent years. Regardless of the source, if three incident monochromatic lights (corresponding to each RGB component) are acquired by the camera, the RGB value of the obtained interference image can be directly applied to Eq. (1). Therefore, the abovementioned calibration curve can be obtained analytically without a reference device. However, as in the case of white light sources, it is impossible to avoid deterioration in accuracy due to diffuse reflection; thus, RGB values are converted into hues^17^. Hue is a value that qualitatively expresses the color aspect obtained from RGB luminance and is often used in combination with parameters, such as saturation and lightness (HSV color space). The hue H is defined from the RGB luminance ($${I}_{r}$$, $${I}_{g}$$, $${I}_{b}$$) using the following equation ($$0$$$$\le H < 2\pi$$)^28^.

3 $$\tan H = \frac{{\sqrt 3 \left( {I_{g} - I_{b} } \right)}}{{2I_{r} - I_{g} - I_{b} }}.$$

If the wavelength dependence of the reflectance $R(x,y)$ is small, it can be ignored. Furthermore, if ${I}_{1}$ in Eq. (2) is identical for all monochromatic light by calibration, it can be substituted in Eq. (3) to obtain the following simple equation.

4 $$\tan H = \frac{{\sqrt 3 \left\{ {\cos \left( {4\pi t_{o} /\lambda_{g} } \right) - \cos \left( {4\pi t_{o} /\lambda_{b} } \right)} \right\}}}{{2\cos \left( {4\pi t_{o} /\lambda_{r} } \right) - \cos \left( {4\pi t_{o} /\lambda_{g} } \right) - \cos \left( {4\pi t_{o} /\lambda_{b} } \right)}}.$$

**Figure 2:**
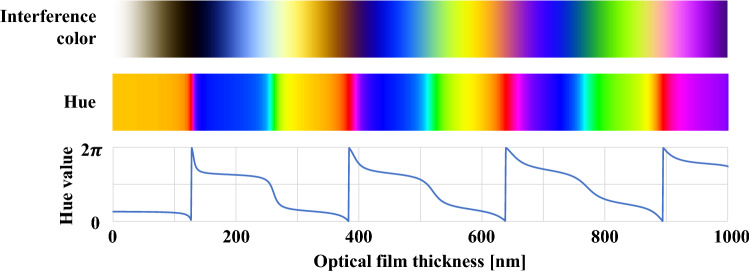
Interference color, hue, and calibration curve of the optical film thickness as a function of the hue for ${\lambda }_{r}$= 600 nm, ${\lambda }_{g}$= 560 nm, and ${\lambda }_{b}$= 470 nm.

Because the hue relies only on the wavelength and optical film thickness of each monochromatic light regardless of the horizontal position, the influence of the decrease in brightness due to diffused reflection can be neglected. Even if $R(x,y)$ changes the magnitude of the RGB luminance, the hue value remains unchanged because the amplitude center and amplitude magnitude in Eq. (2) are equal for each monochromatic light. The gap can be estimated theoretically even for rough surfaces by converting the interference images of three monochromatic lights to optical film thickness via hue conversion. Figure 2 illustrates the calibration curves of the optical film thickness as a function of the hue in the range of 0–1000 nm for the combination of single-wavelength light of ${\lambda }_{r}$= 600 nm, ${\lambda }_{g}$= 560 nm, and ${\lambda }_{b}$= 470 nm^27^ (interference colors are also shown for comparison).

As shown in Eq. (4), the hue value wraps around the optical film thickness in a certain period, and therefore, one hue value corresponds to several candidate values of the optical film thickness. In such cases, the conversion methods based on phase unwrapping^27,29,30^ are used. These methods assume that the change in neighboring pixels is small and adds multiples of $2\pi$ to each folded pixel such that it can be processed as a continuous value. Similarly, if the corresponding optical film thickness is also added, the hue distribution and optical film thickness can be matched on a one-to-one basis.

Because the phase unwrapping continuously refers to the values of neighboring pixels, inaccurate film thickness distributions are obtained in the vicinity of interferograms that contain many areas wherein the optical film thickness suddenly changes. Nevertheless, the period of the irregularity of the roughness curve is considerably smaller than the pixel size, and therefore the variation of the average optical film thickness within a pixel is relatively small. This implies that the phase unwrapping can be applied to rough surface contacts. Contrarily, the roughness curve contains various frequency components and is affected by the shape change of the scale above the order of micrometers—the so-called “waviness curve”—and the amplitude may exceed the fold period of the hue. In addition, when there are macroscopic flaws or foreign matter in the image, the luminance changes only in the vicinity of the flaw. To mitigate the effects of such singular pixels, filtering is often applied during image processing. Typical filters include the Gaussian filter^31^ and median filter^32^, both of which are applied separately to each RGB luminance of the interference image. For normal image processing, the normal filtering process works well; however, for the hue values herein, problems may arise. This is because filtering on RGB luminance breaks the relative relationship between them and causes unnatural wrapping in the hue, which influences the RGB relative relationship. This is particularly problematic when the luminance reduction due to diffuse reflection is large. In this study, we propose a new method of applying a Gaussian filter to the hue distribution instead of the interference image. Because the hue is defined as $0\le H<2\pi$, applying a Gaussian filter directly to the hue results in an inappropriate hue distribution as the folded part between 0 and $2\pi$ is separated, even though it is originally continuous. To avoid this problem, we separate the hue into a sine component, ${H}_{s}$, and a cosine component, ${H}_{c}$, as shown below.

5 $$H_{s} = \sin H\;{\text{and}}\;H_{c} = \cos H.$$

By definition, ${H}_{s}$ and ${H}_{c}$ vary from -1 to 1 and are not wrapped; hence, no discontinuity occurs even if filtering is applied to them. When the filtered values are ${H}_{s}^{^{\prime}}$ and ${H}_{c}^{^{\prime}}$, the following equation can be used to return the filtered hue, ${H}^{^{\prime}}$, without discontinuities.

6 $$\mathrm{tan}{H}^{^{\prime}}={H}_{s}^{^{\prime}}/{H}_{c}^{^{\prime}}.$$

Such hue filtering enables film thickness conversion from hue even if singular pixels are included; however, because the resulting film thickness distribution is less clear owing to the filtering, the original clarity is restored. To restore the original sharpness, the value closest to the film thickness obtained from the hue after filtering should be selected from the film thickness value candidates corresponding to the hue before filtering in each pixel. This process provides an accurate film thickness distribution in most areas.

**Figure 3:**
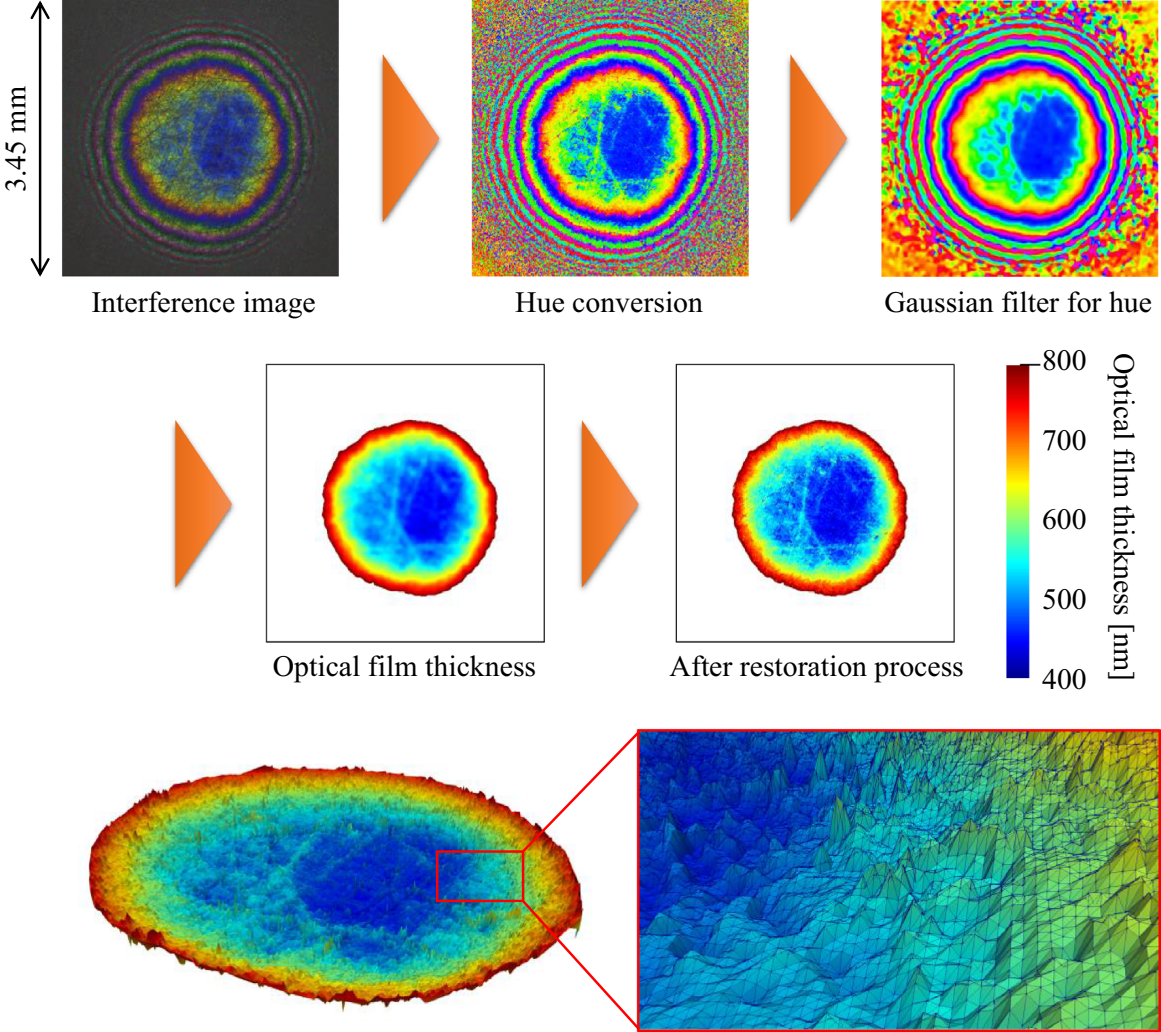
Process of calculating the optical film thickness of a rough contact surface and 3D image of optical film thickness distribution (magnification in the height direction is 500 times).

By combining the three single-wavelength optical interferometry and hue as described above, the effects of diffused reflection are eliminated, and a wide range (${\mathrm{mm}}^{2}$ order) contact state of a rough surface with a square roughness of up to 100 nm can be measured. Figure 3 depicts the process of calculating the optical film thickness from the interference image of a rough contact surface.

## Methods

**Figure 4:**
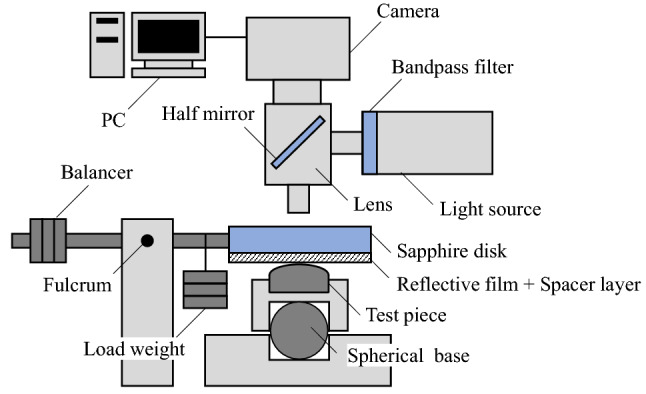
Configuration of test equipment.

We observed the static contact surface between the rough and smooth surfaces using colorimetric interferometry. Figure 4 depicts the configuration of the test equipment. A sapphire disk (modulus of elasticity: 470 GPa^33^) is pressed against the test piece placed on a holder from above at an arbitrary pressing load. The holder can be tilted by a spherical seat and adjusted such that the top of the test piece is in contact with the sapphire surface. The contact surface of the sapphire disk is coated with a Cr semipermeable coating as a reflective film and a SiO_2_ coating as a spacer layer^16–20^. TOYOTA ATF WS (refractive index = 1.47 measured using a precision refractometer [Shimadzu Kalnew KPR-300]) is used as the oil interposed between the sapphire and test piece.

**Figure 5:**
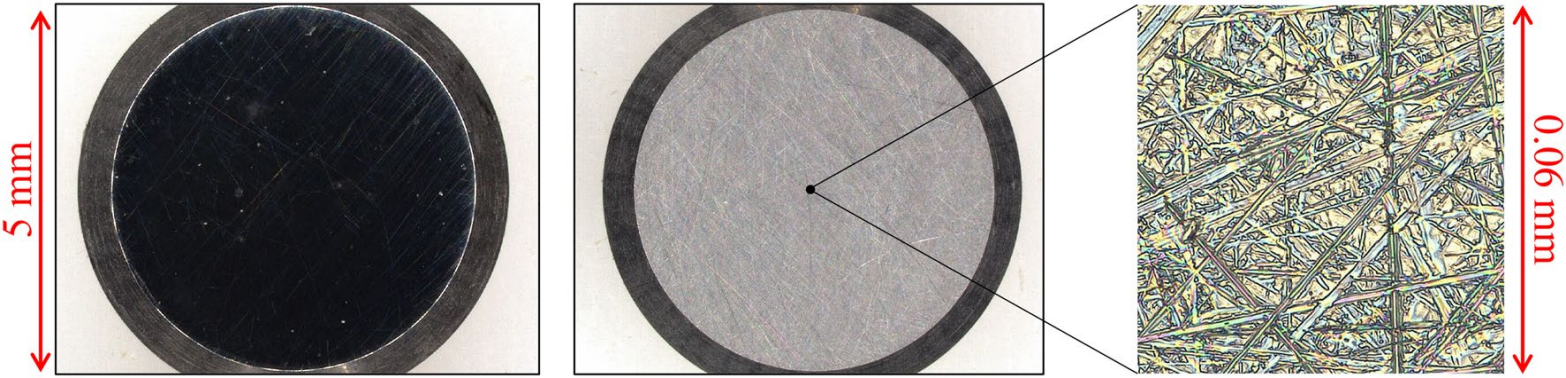
Overall image of test pieces (left: mirror polishing, right: after roughening).

**Figure 6:**
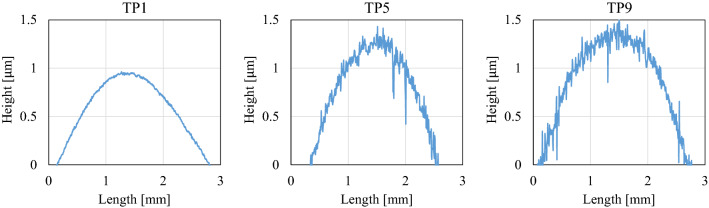
Roughness curve of each test piece.

Each test piece comprises a cylindrical member of 5 mm in diameter (excluding the chamfered part) and SUJ-2 (modulus of elasticity: 208 GPa^34^). The SUJ-2 is processed by a tabletop rotary grinder (Maruto ML-150S) into a gently spherical smooth surface and roughened with sandpaper of #2000 to #10000. Figure 5 shows an example of an entire image and a magnified image of a test piece measured using a digital microscope (Keyence VHX-7000). Table 1 presents the surface parameters ${S}_{\mathrm{q}}$ (root mean square roughness) and ${S}_{\mathrm{R}}$ (spherical curvature radius) of the nine test pieces (TP1 to TP9) used in this study, which are measured using a white interferometric 3D surface profiler (ZYGO NewView 5022). The root mean square roughness represents the value after applying the high-pass filter ($100 \upmu\, \text{m}$) and low-pass filter ($6\,\upmu\, \text{m}$) to the 3D shape data measured at a microscope magnification of 20 × 0.4. The curvature radius represents the radius of the sphere that is fitted using least squares to the 3D shape data measured at a microscope magnification of 5 × 0.5. Figure 6 depicts the cross-sectional curves generated from the 3D shape data for each test piece. Because the curvature radius of a spherical test piece used in such measurements is typically ~ 0.01 m, the size of the contact area is small. However, the test pieces prepared in this study have an extremely large radius of curvature, which enables the observation of the contact state of a considerably wider contact surface in comparison that in with previous studies^16–20^.

**Table 1:** Root mean square roughness and spherical curvature radius of test pieces.

TP No	1	2	3	4	5	6	7	8	9
S_q_ [nm]	6.6	21.0	30.8	49.0	58.3	73.5	78.2	81.0	105.8
S_R_ [m]	0.91	1.28	0.89	0.76	0.41	0.56	0.74	1.12	0.59

## Results

**Figure 7:**
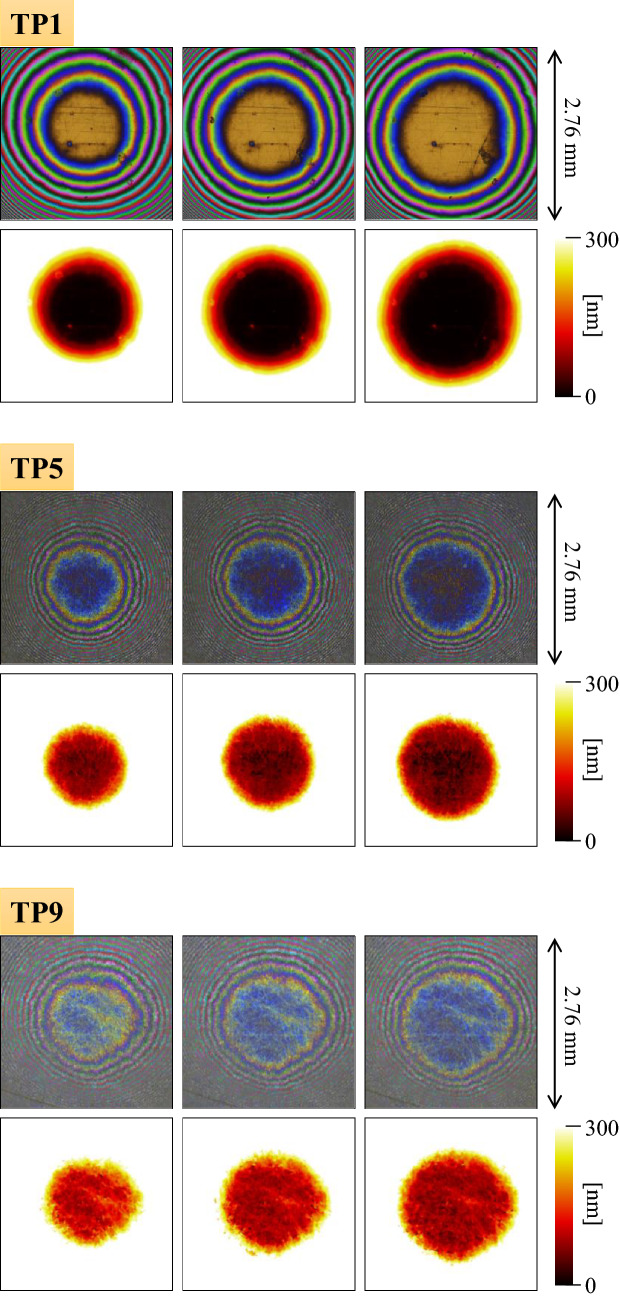
Interference image and mean film thickness distribution of each test piece (from left: pressing loads of 25.4, 50.8, and 88.9 N).

Figure 7 depicts the interference images of the contact surfaces of test pieces 1, 5, and 9 (these are the minimum, intermediate, and maximum root mean square roughness of the test pieces measured in this study) for pressing loads of 25.4, 50.8, and 88.9 N, and the mean film thickness distribution obtained by converting the images. The following equation is used to convert the optical film thickness, ${t}_{o}$, to the actual film thickness, ${t}_{p}$ (unit: nm).

7 $$t_{p} = \left( {t_{o} - 345} \right)/1.47.$$

Here, the value of 345 nm indicates the increase in optical film thickness owing to the reflective film and spacer layer, and the value of 1.47 is the refractive index of oil; the increment is determined via calibration with a smooth test piece. In addition, the RGB values of the interference images are calibrated so that ${I}_{1}$ in Eq. (2) is identical for all monochromatic light. As mentioned above, the mean film thickness value obtained for each pixel (size: $6.9\times 6.9 \,\upmu\, \text{m}$) can be regarded as the value obtained by averaging the gap distribution between the contact surfaces, which are originally distributed in a more complicated manner. Even if local contact occurs within a pixel, the mean film thickness will not be zero. Because there exists almost no difference in the root mean square roughness and roughness shape of test pieces before and after the experiment, we assume that the elastic deformation is dominant in these contacts. Therefore, we consider this phenomenon as pure elastic deformation.

In all the test pieces, a circular expansion of the small film thickness area is observed with increasing load. This is due to the elastic deformation of the spherical surface caused by the contact surface pressure, widely known as Hertz’s contact theory^35^. The mean film thickness decreases with increasing load in the area where contact has already occurred at low loads owing to the elastic deformation of the roughness profile. The reason for the slow decrease is that the macroscopic deformation of the entire sphere occurs simultaneously with the local deformation, which reduces the load concentration. In other words, the rough surface deforms while maintaining its original shape. Only the rough asperities contacting the opposite surface deform elastically and collapse, resulting in a gradual decrease in the overall gap. Meanwhile, the larger the root mean square roughness, the larger the difference in the mean film thickness distribution at each position of the contact area, which can be attributed to the fact that the waviness curve also increases with the increase in the root mean square roughness.

**Figure 8:**
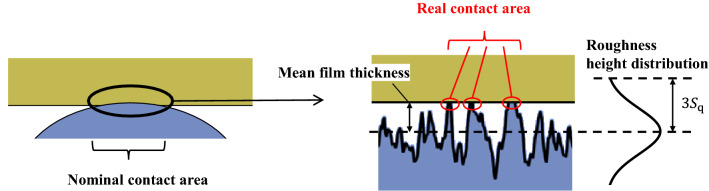
Relationship among mean film thickness, root mean square roughness, and real contact in contact theory.

The proposed method allows for clear observation of the contact state of a wide range of rough surfaces. Next, we examined the measurement results using various scales to study the contact phenomena. First, we determined the effects of the root mean square roughness and pressing load on the nominal contact surface. Although the size of the nominal contact area does not largely affect the frictional force, it is important from the viewpoint of wear and other factors because it indicates the range of contact. In addition, because the nominal contact area is an index that can be compared with theoretical predictions, such as from Hertzian contact analysis, we conducted a quantitative evaluation. As for the definition of the nominal contact area, the contact theory based on statistical theory^11,12,36–40^ is often used. According to reference^38^, if the mean film thickness in a certain area is less than three times the root mean square roughness, it is highly likely that some of the asperities are in contact with the surface; and that the area can be regarded as the nominal contact area (Fig. 8).

**Figure 9:**
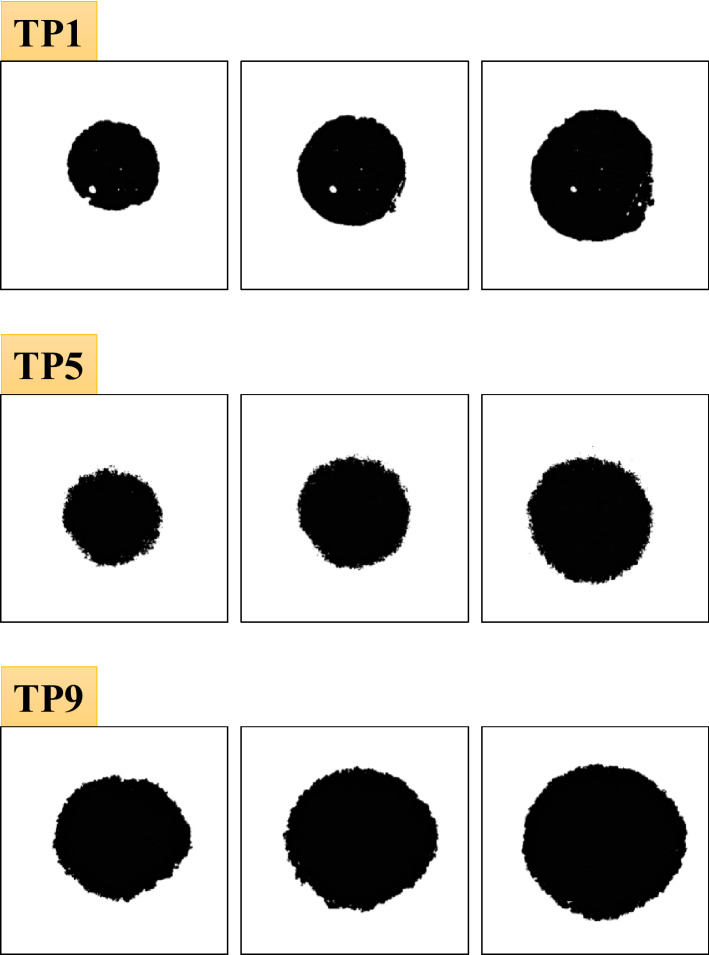
Nominal contact area of each test piece in the contact theory (from left: pressing load of 25.4, 50.8, and 88.9 N).

**Figure 10:**
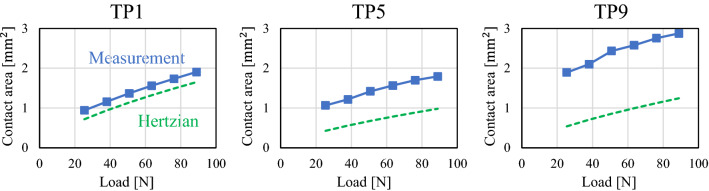
Relationship between nominal contact area and pressing load.

**Figure 11:**
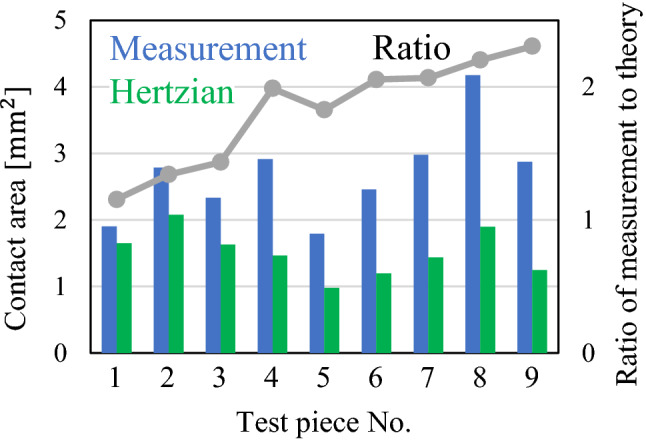
Measured and theoretical contact area for each test piece for pressing load of 88.9 N and ratio of the measurement values to theoretical values.

Based on the abovementioned concept, the pixels whose mean film thickness is less than three times the root mean square roughness of each rough surface are defined as the nominal contact points. Figure 9 depicts the nominal contact area for each test piece and loading condition based on the mean film thickness distribution in Fig. 7. In all test pieces, the nominal contact area is almost circular owing to elastic deformation, and the area boundary is clear. We calculated the values of the nominal contact area; the variation with respect to load for each test piece is depicted in Fig. 10. For comparison, the theoretical values obtained from the Hertzian contact theory^35^ assuming spherical curvature and the equivalent elastic modulus of each test piece are also plotted (Poisson’s ratio of 0.3). Here, the theoretical value differs depending on the spherical curvature radius of each test piece. The contact area of TP1 with low root mean square roughness agrees well with the theoretical value. However, as the root mean square roughness increases with TP5 and TP9, the nominal contact area becomes significantly larger than the theoretical value. Next, Fig. 11 depicts the measured and theoretical values of the nominal contact areas for each test piece for the same load (88.9 N) and the ratio of the measured values to theoretical values. The root mean square roughness increases from TP1 to TP9, indicating that the increase rate grows as the root mean square roughness becomes larger (with the exception of TP4 owing to an elliptical shape). These results indicate that the Hertzian contact theory is valid only for an extremely smooth sphere and deviates significantly from measurements as the root mean square roughness increases. These results are mentioned in detail in Discussion with the contact theory^41,42^.

**Figure 12:**
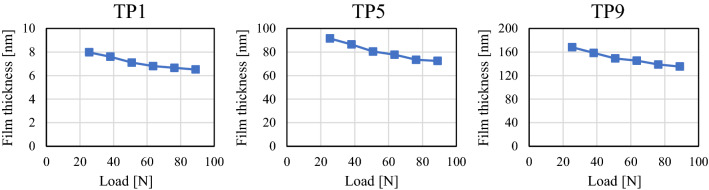
Relationship between contact film thickness and pressing load.

Next, we discuss the dependence of the mean film thickness of the nominal contact area on the root mean square roughness and contact pressure. Strong contact occurs where the mean film thickness is small, resulting in high frictional force and wear during sliding. In addition, if the mean film thickness is small, the traversing of fluids, such as gas and oil, is inhibited; therefore, the amount of mean film thickness is important for evaluating the sealing performance of the contact surface. To facilitate comparison of the contributions of the root mean square roughness and load to the mean film thickness, the average value of the mean film thickness distribution on the nominal contact area is defined as the average contact film thickness. First, to understand the effect of contact pressure, the average value of the contact film thickness is plotted against the pressing load for each test piece in Fig. 12. In all cases, these values decrease gradually with increasing load. The amount of gap decreases owing to the elastic deformation of the roughness asperities as the contact pressure increases.

**Figure 13:**
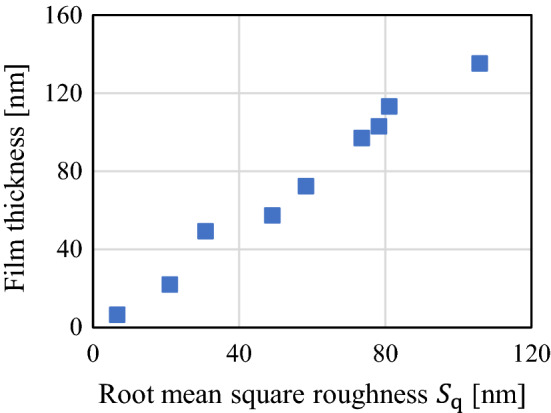
Relationship between contact film thickness and root mean square roughness for pressing load of 88.9 N.

To confirm the effect of the root mean square roughness, the average contact film thickness value is plotted against the root mean square roughness at the same load (88.9 N), and the results are depicted in Fig. 13. These values increase almost proportionally with the root mean square roughness. Moreover, the contact pressure acting on the nominal contact area is different for each test piece even for the same pressing load, because the contact area differs for each test piece. However, the change in the gap size against the contact pressure is considered to be relatively small; thus, the average contact film thickness value for the same contact pressure can be considered to be proportional to the root mean square roughness (at least for values below 100 nm).

## Discussion

**Figure 14:**
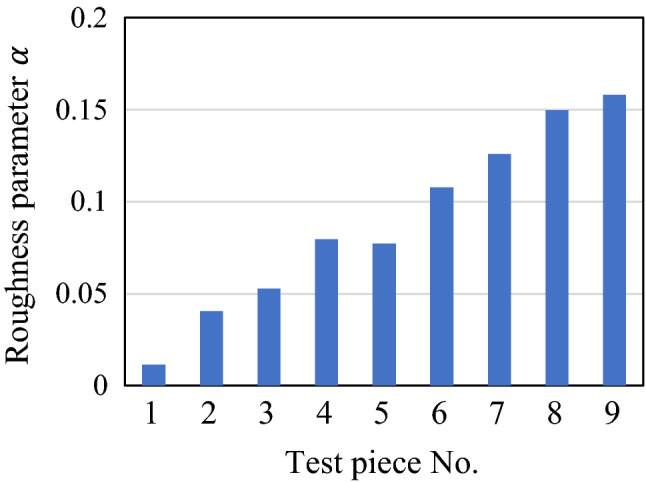
Roughness parameter $\alpha$ for each test piece for pressing load of 88.9 N.

In this section, the experimental results are compared with those obtained from conventional contact theory and computational simulation. We found that the nominal contact area (in mm) may increase by more than twice the size for root mean square roughnesses of the order of 100 nm at most. At first glance, such a phenomenon seems unreasonable, although it has been predicted by both theoretical analysis and simulation. As a concrete example, Greenwood et al.^41,42^ predicted that the contact pressure distribution acting on a nominal contact area of the rough surface would be wider than that of the smooth surface owing to the contact with the rough asperities, resulting in greater elastic deformation and an increase in the radius of the nominal contact circle. The degree of increase was expressed by the following formula (rewritten for consistency with the conditions and annotations herein).

8 $$\alpha = S_{{\text{q}}} S_{{\text{R}}} /a_{0}^{2} ,$$

where ${a}_{0}$ is the radius of the contact circle predicted by the Hertzian contact theory. Figure 14 shows the value of $\alpha$ for each test piece for the pressing load of 88.9 N, and it can be seen that $\alpha$ also increases as the root mean square roughness of the test piece increases. That is, for the same spherical radius of curvature and pressing load, the value of $\alpha$ increases proportionally with the increase in root mean square roughness, such that the nominal contact area is larger than the value predicted theoretically.

The results of this study are compared with Greenwood’s results^41,42^ below. First, it should be noted that the effective contact radius ${a}^{*}$ defined by Greenwood has a very different physical meaning from the nominal contact circle in this study. Specifically, Fig. 1 reported by Greenwood^42^ shows the contact pressure distribution, and it can be seen that the effective contact radius ${a}^{*}$ is much closer to the center of the contact circle than the edge of the distribution. On the other hand, because the nominal contact occurs in the range where even a small amount of contact pressure is applied, the edge of the contact pressure distribution corresponds to the nominal contact radius. For example, according to Fig. 1 reported by Greenwood^42^, the nominal contact radius is about 1.2 times the Hertzian contact radius at $\alpha =0.047$, which indicates that the nominal contact area is about 1.4 to 1.5 times the nominal contact area of Hertzian theory. As shown in Fig. 11, the nominal contact area of TP3 ($\alpha =0.053$) is 1.43 times the contact area of Hertzian theory. Therefore, the results of this study are similar to those by Greenwood's theory. Note that in the original work^42^, it was experimentally confirmed that Greenwood's theory held true for rough surfaces with root mean square roughnesses larger than 100 nm. On the other hand, in this study, it was confirmed that the nominal contact area increased significantly even with a small ${S}_{\mathrm{q}}$ of 6 to 100 nm and that Greenwood's theory could be applied. This is because the test pieces used in this study have larger curvature radii ($>$ 0.4 m) than typical ball test pieces (~ 0.01 m). Using a large curvature radius, it is possible to clearly show that the value of $\alpha$ increases to some extent even if ${S}_{\mathrm{q}}$ is small and that the nominal contact area increases.

In addition, FEM analysis^13^ has also been conducted, and the results support the theoretical prediction of the increase in the contact pressure distribution and nominal contact area. Therefore, our measurement results are reasonable and demonstrate that even a low root mean square roughness may significantly affect the nominal contact area. This observation is crucial for practical use because it implies that the pressure receiving area changes with the root mean square roughness. For example, by increasing the root mean square roughness on the contact surface where fatigue fracture is a concern owing to repeated contact, the surface pressure is dispersed. Furthermore, internal stress may be relaxed and product life may be improved.

## Conclusion

In this study, we developed and verified a method for the direct observation of the contact state to clarify the effect of surface roughness on the contact phenomenon. First, we applied colorimetric optical interferometry to accurately measure the contact state of rough surfaces. Using the interferometric light intensity from three single-wavelength light sources, we calculated the relationship between the optical film thickness and hue, and theoretically demonstrated that the effect of roughness diffuse reflection can be neglected. Furthermore, we proposed a new method that combines the conversion method based on phase unwrapping with the hue filtering process and the restoration process to convert the obtained interferogram image between the contact surfaces into the mean film thickness distribution.

Using the proposed method, we measured the static contact surfaces of spherical test pieces with various root mean square roughness values that were pressed against sapphire and confirmed that a wide range of mean film thickness distributions formed by elastic deformation could be obtained. In particular, as the root mean square roughnesses increased, the measured values of the nominal contact areas became larger than those obtained according to the Hertzian contact theory; this is consistent with the predictions of Greenwood’s contact theory. Namely, it was newly confirmed that contact theory could be applied even for very small ${S}_{\mathrm{q}}$ values of 100 nm or less.

Finally, the advantage of this measurement is that it can simultaneously capture the macroscopic horizontal contact area in the order of millimeters and the microscopic film thickness distribution in the order of nanometers, which is expected to further expand the range of application. In this study, we focused on static contact surfaces; however, this approach can be easily extended to dynamic sliding surfaces, e.g., to perform real-time measurement of film thickness change due to wear. It is also possible to measure various other surface properties, which may significantly contribute to the further elucidation of contact phenomena.
